# Ultrashort Cationic Lipopeptides and Lipopeptoids Selectively Induce Cytokine Production in Macrophages

**DOI:** 10.1371/journal.pone.0054280

**Published:** 2013-02-04

**Authors:** Brandon Findlay, Neeloffer Mookherjee, Frank Schweizer

**Affiliations:** 1 Department of Chemistry, University of Manitoba, Winnipeg, Manitoba, Canada; 2 Department of Internal Medicine and Immunology, Manitoba Centre for Proteomics and Systems Biology, University of Manitoba, Winnipeg, Manitoba, Canada; 3 Department of Medical Microbiology, University of Manitoba, Health Sciences Centre, Winnipeg, Manitoba, Canada; University of São Paulo, Brazil

## Abstract

A series of ultrashort lipopeptides and lipopeptoids were tested for their ability to induce cytokine production in macrophages. Fourteen compounds were found to strongly induce production of chemokines Groα and IL-8, with a structural bias that was absent from previous antibacterial activity investigations. Compounds based on LysGlyLys and *N*LysGly*N*Lys sequences did not induce cytokine production, whereas those based on LysLysLys and *N*Lys*N*Lys*N*Lys were active only when linked to a lipid tail at least sixteen carbons long. Three lipopeptides induced high levels of IL-8 production, above that of equivalent concentrations of cathelicidin LL-37, while no compound induced production of the pro-inflammatory cytokine TNF-α at or below 100 µM. Two compounds, peptoids C16OH-*N*Lys*N*Lys*N*Lys and C16OH-*N*Har*N*Har*N*Har, were selective for IL-8 production and did not induce TNF-α or IL-1β. These compounds may prove beneficial for in vivo treatment of infectious disease, with improved bioavailability over LL-37 due to their protease-resistant scaffold.

## Introduction

It is well established that the prevalence of antibiotic resistance is rising in pathogenic bacteria, in part due to selective pressure from human antibiotic use and from the use of antibiotics as growth promoters in livestock [1], [2]. Efforts to develop new antibiotics and antibiotic scaffolds have met with mixed success, due to reduced industry investment and a number of complications arising from high-throughput screening of bacterial targets [3]. The urgent need for new antibiotics has driven research into new scaffolds and unconventional modes of action, with the field of cationic antimicrobial peptides (CAMPs) of particular interest [4]. First isolated from amphibians like *Xenopus laevis* [5], CAMPs are a rich class of structurally diverse antimicrobials found throughout the plant and animal kingdoms. Unlike classical antibiotics, which derive their activity through inhibition of specific enzymes or processes, CAMPs largely interact with the cell through non-specific interactions driven by a mix of electrostatic and hydrophobic effects [6]. Early researchers were particularly interested in the effect of CAMPs on bacterial and mammalian membranes, as many compounds were found to form membrane pores at sufficiently high concentrations and it was thought that a membrane-specific mode of action had reduced potential for resistance development [7], [8].

These initial investigations into pore formation eventually led to a more nuanced view of CAMPs [9]. Examining CAMPs with high levels of antibacterial activity and low mammalian toxicity revealed that at their minimal inhibitory concentration (MIC) they were interfering with either internal cellular processes or membrane proteins, not predominantly with the bacterial membrane [4], [10]. As efforts to optimize the more membrane-active peptides have been stymied by these peptides’ ability to disrupt or lyse eukaryotic cells at concentrations generally only slightly above that of bacterial membranes [11], much of the research in recent years has focused on understanding the interaction between CAMPs and their non-membrane targets [4], [12], [13].

The human cathelicidin LL-37 has been a subject of particular interest, due to its widespread expression in human immune and epithelial cells. First discovered through its LPS binding ability [14], LL-37 is strongly antibacterial *in vitro* but has little antibacterial activity under physiological conditions [15]. Instead, LL-37 appears to exert its antibacterial effect through modulation of the immune system [16], [17]. Consistent with this, altered LL-37 expression has been linked to both auto-immune disorders and increased susceptibility to bacterial infection [18]. LL-37 promotes wound healing [18], recruits leukocytes to the site of infection by acting as a direct chemoattractant or by inducing chemokine production, activates local dendritic cells and T-cells for clearing of invasive bacteria, and exhibits selective anti-inflammatory effects [17]. Modification of another immunomodulatory peptide, the bovine bactenacin, created IDR1, which has protective effects in a mouse infection model, despite lacking direct antibacterial activity [4], [19]. Further library screening of bactenacin derivatives led to the more active IDR-1002 [20], while a proprietary compound, the pentapeptide IMX942, is currently entering phase 2 clinical trials [21].

To date, research into the immunomodulatory properties of CAMPs has focused on analogues of natural host defence peptides (HDPs), paring down the length of the peptide to reduce production costs while retaining a modified subsection for receptor binding [19]. As natural immunomodulators like LL-37 act in part through low-affinity binding to chemotaxis receptor FPRL1 and to several intracellular receptors [22], [23], we hypothesized that compounds which mimic the physiochemical properties but not the sequence of HDPs may demonstrate improved activity, while allowing for incorporation of a protease-resistant scaffold. Protease resistance was desirable, as bacterial resistance to LL-37 can be readily conferred by metalloprotease secretion, increasing virulence [24]. Recent reports confirmed this early hypothesis [25], demonstrating that protease-resistant synthetic mimics of antibacterial peptides based on a cationic arylether scaffold cause immunomodulatory responses.

Previous work by us and others has shown that for ultrashort lipopeptides and lipopeptoids only three amino acid residues and a hydrophobic lipid tail is required for efficient killing of a wide variety of bacteria *in vitro*.[26]–[28] Antibacterial activity was related to both peptidic sequence and length of the hydrophobic tail, with our most active lipopeptides and lipopeptoids unfortunately demonstrating significant toxicity towards mammalian red blood cells at slightly above their effective antibacterial concentrations. In lipopeptides this toxicity could be sharply reduced by adding a polar hydroxyl group to the terminus of the lipid tail [27], but these amphiphiles had little antibacterial activity.

With their large lipid tail ultrashort lipopeptides bear little similarity to known HDPs, and have shorter peptide sequences than the innate defence regulator peptides currently in clinical development. Like LL-37, *in vitro* they disrupt the bacterial membrane [26], [29], and we hypothesized that they could also modulate the immune system. As achiral molecules the lipopeptoids are further from the structure of current innate defence regulator peptides, but their naturally protease-resistant backbone offers several key advantages (*vide supra*) [30]. The purpose of this study was to assess the ability of a series of ultrashort lipopeptides and lipopeptoids to induce production of chemokines Groα and IL-8 in human macrophage-like THP-1 cells. Macrophage-like THP-1 cells elicit cellular responses similar to peripheral blood-derived mononuclear cells in the presence of host defence peptides [31], [32], while Groα and IL-8 play a critical role in leukocyte recruitment to the site of infections, enhancing bacterial clearance [33]. We further screened active compounds for the production of pro-inflammatory cytokines TNF-α and IL-1β. Compounds which selectively induce chemokine production without inducing pro-inflammatory cytokine TNF-α may be useful in antibacterial therapy.

## Results and Discussion

**Figure 1:**
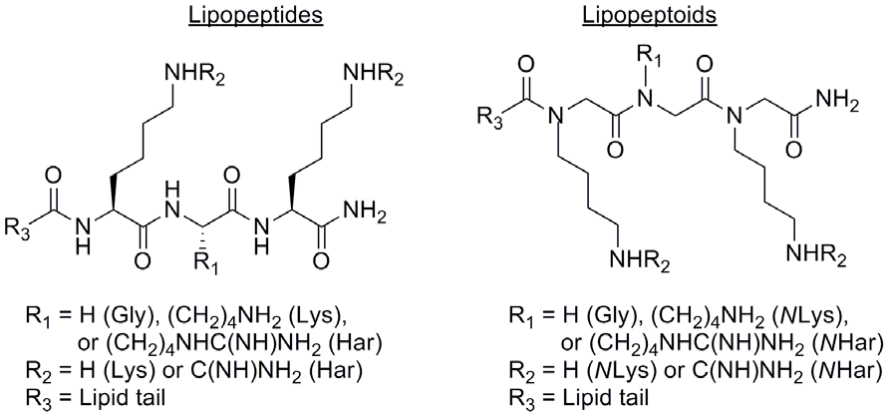
Structures for the cationic amphiphiles used in this study. Har = homoarginine; *N*Lys = lysine peptoid; *N*Har = homoarginine peptoid.

To display antibacterial activity lipopeptides and lipopeptoids require an overall cationic charge and long lipid tail, for attraction to the negatively charged bacterial outer membrane and insertion into the hydrophobic membrane core [26]. Our compound synthesis was biased towards these properties (General Chemical Procedures, Supplementary Materials), with LysGlyLys or LysLysLys based amphiphiles (*N*LysGly*N*Lys and *N*Lys*N*Lys*N*Lys for the lipopeptoids) and lipid tails eleven to twenty carbons in length (Figure 1, Table 1, Table S1). Aware of the potential cytotoxicity of amphiphiles with large hydrophobic tails, a terminal hydroxy group was added to the C16 tails to disturb the classic amphiphilic nature of lipopeptides **6** and **10** and lipopeptoids **17** and **20**. Increasing the strength of the cationic charge has been found to improve antibacterial activity [28], and so each of the LysLysLys and *N*Lys*N*Lys*N*Lys compounds was mirrored by a homoarginine analogue (Har or *N*Har), to determine if immunomodulatory properties would be similarly enhanced. Amphiphile selection was roughly divided between lipopeptides and lipopeptoids, to assess the impact of chirality and conformation on activity.

**Table 1:** Cationic amphiphiles in this study.

	Lipopeptides^a^		Lipopeptoids^a^
1	C16-LysGlyLys-NH_2_	12	C11-*N*LysGly*N*Lys-NH_2_
2	C16OH-LysGlyLys-NH_2_	13	C16-*N*LysGly*N*Lys-NH_2_
3	C20-LysGlyLys-NH_2_	14	C11-*N*Lys*N*Lys*N*Lys-NH_2_
4	C11-LysLysLys-NH_2_	15	C16-*N*Lys*N*Lys*N*Lys-NH_2_
5	C16-LysLysLys-NH_2_	16	C16OH-*N*Lys*N*Lys*N*Lys-NH_2_
6	C16OH-LysLysLys-NH_2_	17	C20-*N*Lys*N*Lys*N*Lys-NH_2_
7	C20-LysLysLys-NH_2_	18	C11-*N*Har*N*Har*N*Har-NH_2_
8	C11-HarHarHar-NH_2_	19	C16-*N*Har*N*Har*N*Har-NH_2_
9	C16-HarHarHar-NH_2_	20	C16OH-*N*Har*N*Har*N*Har-NH_2_
10	C16OH-HarHarHar -NH_2_	21	C20-*N*Har*N*Har*N*Har-NH_2_
11	C20-HarHarHar-NH_2_		LL-37^b^

a Trifluoroacetate salt;

b LLGDFFRKSKEKIGKEFKRIVQRIKDFLRNLVPRTES-OH.

**Figure 2:**
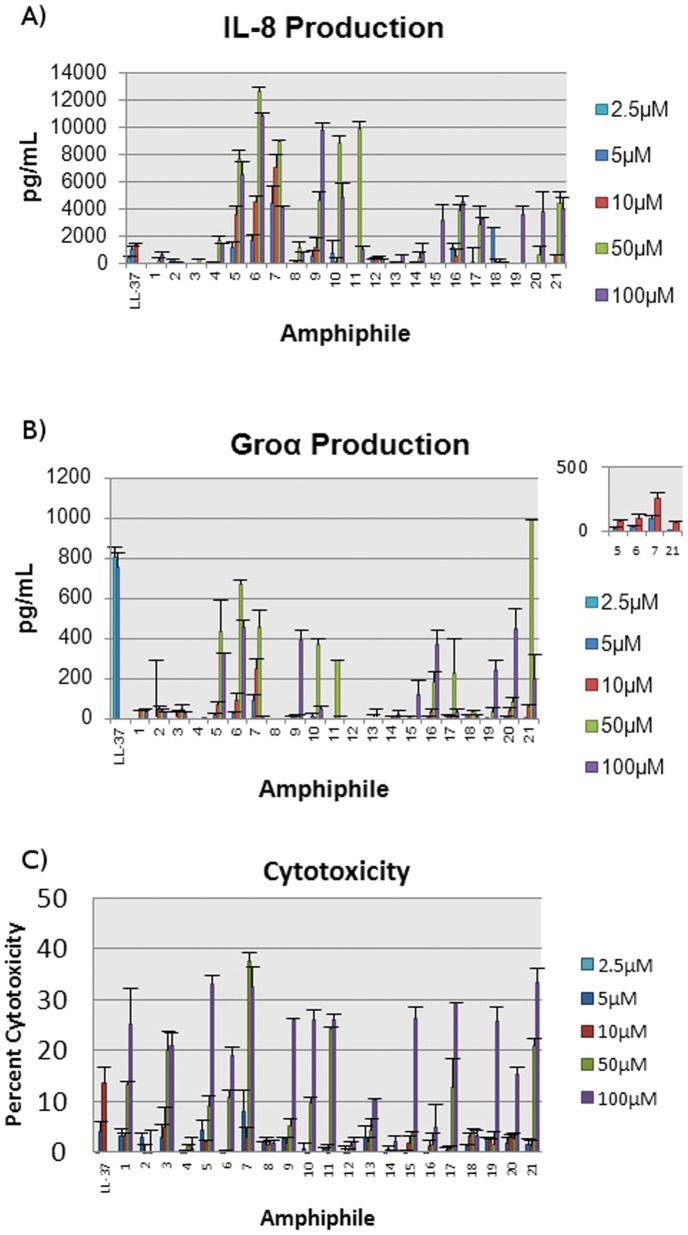
Cytokine production and LDH release by human macrophage-like THP-1 cells following incubation with amphiphiles 1–21. A) IL-8 production. TC supernatants were monitored for IL-8 production by ELISA, and results were recorded in pg/mL. B) Groα production, in pg/mL. Inset: Expanded values for amphiphiles 5–7 and 21 at 5 µM and 10 µM. C) Cytotoxicity following incubation with amphiphiles 1–21. TC supernatants were monitored for LDH release as a measure of cellular toxicity, and results shown represent percent cytotoxicity over un-stimulated cells. All studies were performed in two independent biological replicates with two technical replicate each, with the data here presented as the mean plus standard error of the mean (sem) and with LL-37 data included as a positive control.

Prior to testing, human monocytic THP-1 cells were differentiated to plastic-adherent macrophage-like THP-1 cells as previously described [31]. The cells were rested for twenty-four hours, then exposed to the amphiphiles of interest for twenty-four hours. Cell free TC supernatants were monitored for the production of chemokines Groα and IL-8 by ELISA as previously described [31], [34]. The constitutive background level of chemokine Groα was 6.6±1.8 pg/ml, and that of IL-8 was 1.7±0.16 ng/ml. Encouragingly, the majority of compounds were strong inducers of both IL-8 and Groα and increased chemokine levels above background (Figure 2). The lipopeptides (**1–11)** were more active than their corresponding lipopeptoids (**12–21)**, despite little difference in cytotoxic behaviour. The three amphiphiles with a LysLysLys peptide sequence and lipid tail at least sixteen carbons in length, **5–7**, were especially strong inducers of IL-8, increasing the concentration to 5–8 ng/mL (p<0.05) above control at 10 µM of lipopeptide, and up to 13 ng/mL (p<0.01) above control at 50 µM (Figure 2). These amphiphiles compared favourably to LL-37 (1.3 ng/mL of IL-8 above control at 10 µM), despite their brief peptide sequence. This activity was not shared by the LysGlyLys lipopeptides **2** and **3**, which failed to significantly increase IL-8 production at any of the concentrations tested. Guanidinylation did not improve IL-8 production at low lipopeptide concentrations, though amphiphiles **10–11** did induce up to 10 ng/mL (p<0.05) of IL-8 at 50 µM, suggesting a minimum lipopeptide concentration is required for IL-8 production. All lipopeptides with lipid tails shorter than sixteen carbons were unable to induce either IL-8 or Groα at concentrations up to 100 µM (Figure 2, Figure S1).

The lipopeptides were less effective at inducing Groα production, though 50 µM of amphiphile **6** compared favourably to 2.5 µM of LL-37 (670 pg/mL vs 800 pg/mL, respectively). Lipopeptoid **21** was the strongest inducer of Groα among our amphiphiles, causing 996±4 pg/mL (p = 0.003) of Groα production at 50 µM (Figure 2). This peptoid is composed of a twenty carbon lipid tail and *N*Har*N*Har*N*Har sequence, and along with most of the lipopeptoids tested induced 6±0.8 ng/mL (p<0.05) of IL-8 at 50 and 100 µM amphiphile concentrations. The high level of Groα production at 50 µM did not extend from similar productions at lower concentrations, with peptoids **15–17** significantly weaker than amphiphiles **5–7** at 5 µM and 10 µM. Similar to the results with the lipopeptides, only lipopeptoids based on the *N*Lys*N*Lys*N*Lys sequence (**14–21**) were able to induce IL-8 or Groα production; *N*LysGly*N*Lys peptoids **12** and **13** were inactive.

Chemokine production by the lipopeptides and lipopeptoids was largely unaffected by disrupting the hydrophobic nature of the lipid tail with a terminal alcohol moiety. Little difference was observed between C16-LysLysLys (**5**) and C16OH-LysLysLys (**6**) at low concentrations, and amphiphile **6** led to the highest IL-8 production (12.5±0.5 ng/ml, p = 0.006) at high amphiphile concentration (50 µM). This is in contrast to antibacterial activity, which has been found to be sharply reduced by the presence of a terminal alcohol function [27]. As the antimicrobial activity of lipopeptides is mediated through non-specific membrane interactions [26], this discrepancy suggests that chemokine induction by our compounds is independent of membrane binding.

This is further supported by the lack of activity of amphiphiles **1–3** in contrast to **5–7**. At a concentration of 50 µM, C16-LysGlyLys (**1**) induced 0.2 ng/mL production of IL-8 above control values, while C16-LysLysLys (**5**) increased IL-8 production to 7.7±0.6 ng/mL, p = 0.02 (Figure 2), despite comparable antibacterial activities against both Gram negative and Gram positive bacteria [27]. The significant loss in cytokine production observed between low concentrations of lipopeptides containing LysLysLys and HarHarHar sequences (compounds **5–7** and **9–11,** respectively) also suggests a sequence specific effect, though with the lipopeptoids this difference was not observed.

In contrast, cytotoxicity of the amphiphiles was independent of the peptidic sequence and correlated well with previously published antimicrobial activities and with lipid tail length (Figure 2, Table S2) [27], [28]. At high concentrations the amphiphiles appear able to disrupt the cellular membrane of macrophage-like cells, as cytotoxicity was evaluated through the release of the cytosol-localized protein lactate dehydrogenase (LDH). Our amphiphiles were broadly non-toxic at 5 µM and 10 µM, though the majority caused greater than 15% cytotoxicity at 100 µM; with amphiphiles **3**, **7**, **11** and **21** equally toxic at 50 µM. The high toxicity of amphiphile **21** may limit chemokine induction at high concentrations, explaining the sharp decrease in Groα production as peptoid concentration increases from 50 µM to 100 µM (1000 pg/mL vs 200 pg/mL).

Encouraged by the ability of our amphiphiles to induce chemokines Groα and IL-8, we examined the effect of the active amphiphiles on production of pro-inflammatory cytokines IL-1β and TNF-α. Binding of microbial lipopeptides Pam_3_CSK_4_ and MALP-2 to TLRs strongly induces the production of pro-inflammatory cytokines TNF-α and IL-1β, among others [35]. Both of these bacterial lipopeptides have multiple lipid tails (three and two, respectively), but synthetic analogues with only a single lipid tail have recently been produced [36], suggesting that the activity of amphiphiles **1–21** may stem from binding to TLRs [37]. Pro-inflammatory cytokines help combat infection, but inappropriate or amplified induction of these cytokines – especially TNF-α – leads to chronic inflammatory disorders such as rheumatoid arthritis, inflammatory bowel disease and psoriasis [38], reducing the utility of inflammatory compounds in infectious disease therapy.

**Figure 3:**
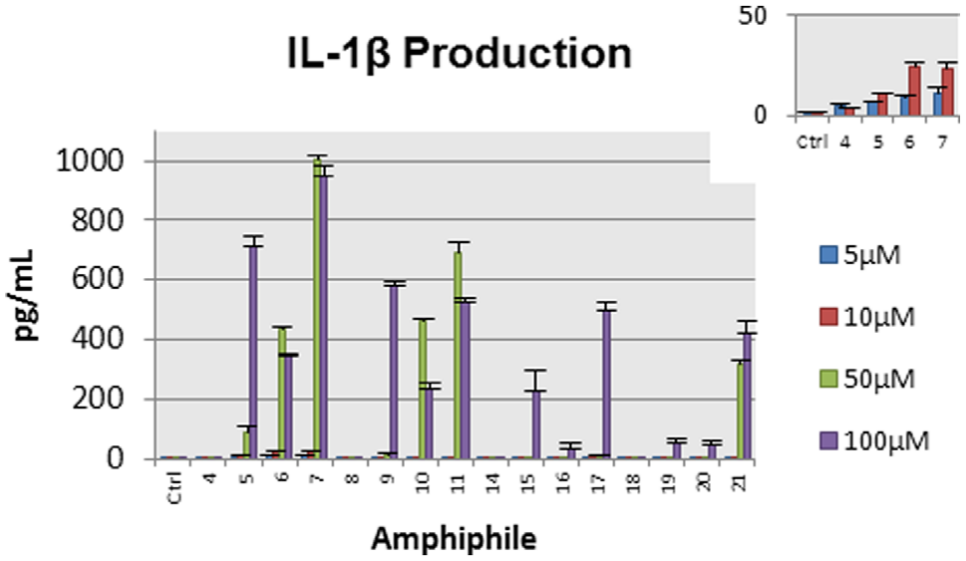
IL-1β production. Human macrophage-like THP-1 cells were exposed to amphiphiles **4–11** and **14–21**, for twenty-four hours. TC supernatants were monitored for IL-1β by ELISA, with results shown in pg/mL. Studies were performed in two independent biological replicates with two technical replicate each, with the data here presented as the mean plus sem. Inset: Expanded results for the negative control and amphiphiles **4–7** at 5 µM and 10 µM.

In contrast to the previously published non-proteogenic immunomodulators [25], none of our compounds induced TNF-α production at any of the concentrations tested (Figure S2), suggesting that the immunomodulatory activity of our compounds is not mediated by engagement of innate immune receptors such as TLRs. However, low concentrations of **5–7** (10 µM) were able to induce up to 25 pg/mL (p<0.06) of IL-1β (Figure 3). Moving from 10 µM to 50 µM of **7** increased the observed IL-1β concentration over forty-fold (p = 0.01), suggesting a threshold peptide concentration was required for strong induction of IL-1β. It was previously demonstrated that the natural HDP LL-37, but not synthetic peptide IDR-1, acts synergistically with IL-1β to induce the chemokine IL-8 [31]. Induction of IL-1β may contribute in part to the high induction of IL-8 observed with our lipopeptides, as the spike in IL-1β production occurs over the same concentration as the increase in IL-8 production by amphiphiles **9–11** and **21.**

### Conclusion

We have shown that ultrashort lipopeptides and lipopeptoids are able to induce selective cytokine production in human macrophages, despite little structural similarity to any known host defence peptide or CAMP. A lipid tail at least sixteen carbons long was required for activity, though the immunomodulatory effect of these compounds does not appear to be related to their effects on the bacterial membrane. Compounds with no appreciable antibacterial activity (MIC 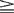
128 µg/mL) were able to strongly induce the production of chemokines IL-8 and Groα at sub-cytotoxic concentrations, though the strongest inducer of Groα, C20-*N*Har*N*Har*N*Har (**21**), also has moderate antibacterial activity *in vitro* (MIC ≤16 µg/mL, Gram positive strains) [28].

The LysLysLys series of peptides induced the greatest IL-8 production, even above that of LL-37. However, these compounds also caused a moderate amount of IL-1β production at the same concentration (10 µM, 25 pg/mL). Lipopeptoids were in general less active, though the peptoid C20-*N*Har*N*Har*N*Har was the strongest inducer of Groα, and a moderate inducer of IL-8. High concentrations of amphiphiles can result in IL-1β production, which may be synergistic in inducing the chemokine IL-8 [31], [39]. None of the compounds induced TNF-α at any concentration tested, suggesting that cytokine production is not mediated by binding to TLR1:TLR2 or TLR6:TLR2 heterodimers [35]. The exact mode of action for these amphiphiles is unknown, though at least two different cytokine expression profiles were observed. The lipopeptoids C16OH-*N*Lys*N*Lys*N*Lys (**16**) and C16OH-*N*Har*N*Har*N*Har (**20**) were of particular interest, as they were moderate inducers of Groα and IL-8 but did not induce either IL-1β or TNF-α production. These compounds are naturally protease resistant, and may be appealing leads for further discovery of highly selective immunomodulation-based infectious disease therapeutics.

## Materials and Methods

### Chemical Synthesis

Lipopeptides and lipopeptoids were prepared according to previously established techniques [26]–[28]. In brief, peptides were synthesized as C-terminal amides on Rink amide MBHA resin, using 9-fluorenylmethoxycarbonyl/t-butylcarbamate (Fmoc/Boc) chemistry. Amino acids were coupled to the resin with O-(Benzotriazol-1-yl)-N,N,N′,N′-tetramethyluronium tetrafluoroborate (TBTU) activation in dimethylformamide (DMF), with the lysine R-groups protected via Boc. Lipid tails were also added with TBTU, and the completed peptides were cleaved from the resin with trifluoroacetic acid (TFA). Peptides were purified via passage through C18-functionalized silica at 3 PSI. Lipopeptoids were also prepared on Rink amide MBHA resin. Bromoacetic acid was added to the growing chain after activation with diisopropylcarbodiimide (DIC), and the alkyl bromine atom was then displaced by tert-butyl 4-aminobutylcarbamate in N-methyl-2-pyrrolidinone (NMP). Lipid tails were attached via activation with TBTU in DMF, and cleavage and subsequent purification were effected similar to the lipopeptides. Guanidinylation was the result of treatment of the lipopeptides and lipopeptoids with N,N-diBoc-N-triflylguanidine and subsequent deprotection with TFA [40]. For further details on the chemical synthesis please see the Supporting Information.

### Cytokine Measurements and Cytotoxicity

Human monocytic THP-1 (ATCC® TIB-202) cells were cultured in RPMI-1640 medium containing 2 mM L-glutamine, 1 mM sodium pyruvate, supplemented with 10% (v/v) FBS and maintained in a humidified incubator at 37°C and 5% CO_2_ as previously described [31]. The cells were differentiated into plastic adherent macrophage-like cells with phorbol 12-myristate 13-acetate (Sigma-Aldrich) and rested for an additional 24 before stimulations as previously described [31]. Macrophage-like THP-1 cells were stimulated with the different compounds for 24 hr. TC supernatants were centrifuged at 1500×g for 5–7 min to obtain cell-free samples and aliquots were stored at −20°C until further use. Cellular cytotoxicity was evaluated by monitoring the release of lactate dehydrogenase employing a colorimetric detection kit (Roche Diagnostics). Production of chemokines Groα and IL-8 were monitored in the TC supernatants by ELISA employing human DuoSet (R&D Systems Inc.) as per the manufacturer’s instructions. Production of pro-inflammatory cytokines TNF-α and IL-1β were monitored in the TC supernatants using specific antibody pairs from eBioscience, Inc., as per the manufacturer’s instructions. The concentration of the cytokines or chemokines in the TC supernatants was evaluated by establishing a standard curve with serial dilutions of the recombinant human cytokines or chemokines [31].

### Statistical Analysis

ELISA results were obtained from two biological replicates with two technical replicates each and statistical analyses was performed using Student paired t-test. Error bars in the graphs represents standard error of the mean, and a p-value of less that 0.05 was considered to be statistically significant.

## Supporting Information

Figure S1 Immunological properties of the compounds presented in table S1. Human macrophage-like THP-1 cells were exposed to amphiphiles **S1–S7** for twenty-four hours. TC supernatants were monitored for A) IL-8 production and B) Gro-α production by ELISA. IL-8 production is shown after subtraction of constitutive background levels found in un-stimulated control cell. C) LDH release was monitored in the TC supernatants as an indicator of cellular cytotoxicity. Results shown represent percent cytotoxicity over un-stimulated cells. Studies were performed in two independent biological replicates with two technical replicate each, with the data here presented as the mean plus standard error of the mean (sem).
(TIFF)

Figure S2 TNF-α production by human macrophage-like THP-1 cells following incubation with amphiphiles **1–21**. TC supernatants were monitored for cytokine production via ELISA, and results are reported in pg/mL. All studies were performed in two independent biological replicates with two technical replicates each.
(TIFF)

Table S1 Compound sequences. Sequence information for seven lipopeptides or lipopeptoids that displayed little to no activity.
(DOCX)

Table S2 Antimicrobial activity of select amphiphiles, derived from previous research. Information on compounds **4–7** and **21** is presented, with data from previous studies converted into µM.
(DOCX)

Supporting Information S1 (DOC)
