# Supplementary figures and images for: Ultrashort Cationic Lipopeptides and Lipopeptoids Selectively Induce Cytokine Production in Macrophages
Source: PLoS One. 2013 Feb 4;8(2):e54280. doi: 10.1371/journal.pone.0054280 (PMC3563528; doi:10.1371/journal.pone.0054280)

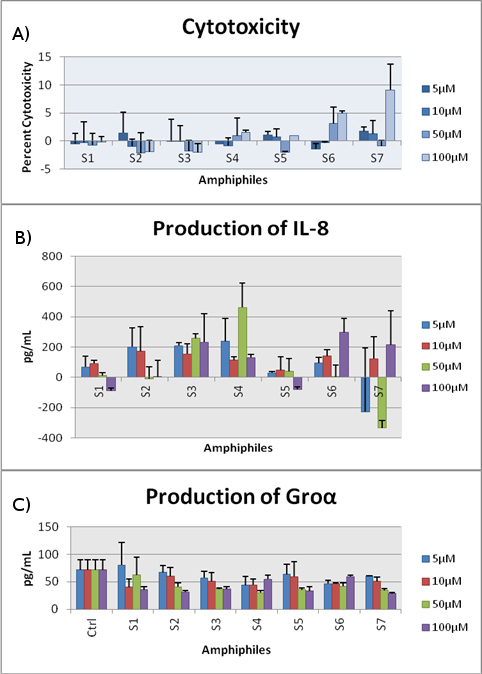

Supplement: Figure S1 — Immunological properties of the compounds presented in table S1. Human macrophage-like THP-1 cells were exposed to amphiphiles S1–S7 for twenty-four hours. TC supernatants were monitored for A) IL-8 production and B) Gro-α production by ELISA. IL-8 production is shown after subtraction of constitutive background levels found in un-stimulated control cell. C) LDH release was monitored in the TC supernatants as an indicator of cellular cytotoxicity. Results shown represent percent cytotoxicity over un-stimulated cells. Studies were performed in two independent biological replicates with two technical replicate each, with the data here presented as the mean plus standard error of the mean (sem). (TIFF) [file pone.0054280.s001.tiff]

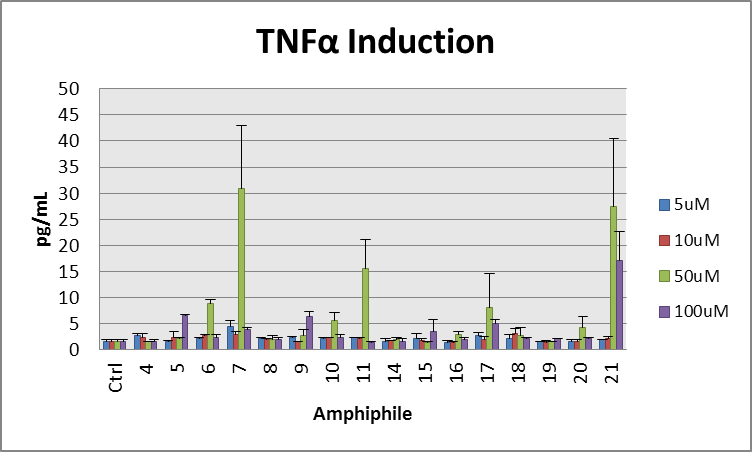

Supplement: Figure S2 — TNF-α production by human macrophage-like THP-1 cells following incubation with amphiphiles 1–21. TC supernatants were monitored for cytokine production via ELISA, and results are reported in pg/mL. All studies were performed in two independent biological replicates with two technical replicates each. (TIFF) [file pone.0054280.s002.tiff]
