# Supplementary material for: Ultrashort Cationic Lipopeptides and Lipopeptoids Selectively Induce Cytokine Production in Macrophages
Source: PLoS One. 2013 Feb 4;8(2):e54280. doi: 10.1371/journal.pone.0054280 (PMC3563528; doi:10.1371/journal.pone.0054280)
Supplement: Table S1 — Compound sequences. Sequence information for seven lipopeptides or lipopeptoids that displayed little to no activity. (DOCX) [file pone.0054280.s003.docx]

|  | **Amphiphile** | **Molecular Mass** |
| --- | --- | --- |
|  | **Lipopeptides**^a^ |  |
| S1 | **C7-LysGlyLys-NH_2_** | 670.64 |
| S2 | **(CH_3_)_2_(CH_2_)_6_COO-LysGlyLys-NH_2_** | 712.72 |
| S3 | **(CF_3_)_2_(CF_2_)_4_(CH_2_)_2_COO-LysGlyLys-NH_2_** | 982.57 |
| S4 | **CF_3_(CF_2_)_5_(CH_2_)_2_COO-LysLysLys-NH_2_** | 117.72 |
| S5 | **CF_3_(CF_2_)_3_(CH_2_)_2_COO -HarGHar-NH_2_** | 916.64 |
|  | **Lipopeptoids**^a^ |  |
| S6 | **CF_3_(CF_2_)_7_(CH_2_)_2_COO-NlysGNlys-NH_2_** | 1032.59 |
| S7 | **C14-NlysNlysNlys-NH_2_** | 953.97 |
