# Supplementary material for: Ultrashort Cationic Lipopeptides and Lipopeptoids Selectively Induce Cytokine Production in Macrophages
Source: PLoS One. 2013 Feb 4;8(2):e54280. doi: 10.1371/journal.pone.0054280 (PMC3563528; doi:10.1371/journal.pone.0054280)
Supplement: Table S2 — Antimicrobial activity of select amphiphiles, derived from previous research. Information on compounds 4–7 and 21 is presented, with data from previous studies converted into µM. (DOCX) [file pone.0054280.s004.docx]

| Sample: | **4** | **5** | **6** | **7** | **21** |
| --- | --- | --- | --- | --- | --- |
| *S.aureus ATCC 29213* | 561^a^ | 8.1 | 128 | 7.7 | 6.9 |
| MRSA ATCC 33592 | >561 | 4.1 | 128 | 7.7 | 14 |
| MSSE 81388 CANWARD 2008 | 281 | <0.25 | 128 | 7.7 | 6.9 |
| MRSE (CZ >32) CAN-ICU 61589 | 281 | <0.25 | 128 | 7.7 | 6.9 |
