# Supplementary material for: Ultrashort Cationic Lipopeptides and Lipopeptoids Selectively Induce Cytokine Production in Macrophages
Source: PLoS One. 2013 Feb 4;8(2):e54280. doi: 10.1371/journal.pone.0054280 (PMC3563528; doi:10.1371/journal.pone.0054280)
Supplement: Supporting Information S1 — (DOC) [file pone.0054280.s005.doc]

**Supporting Information**

# Ultrashort Cationic Lipopeptides and Lipopeptoids Selectively Induce Cytokine Production in Macrophages.

Brandon Findlay, Neeloffer Mookherjee*, and Frank Schweizer*

*Departments of Chemistry, Internal Medicine and Medical Microbiology, University of Manitoba, Winnipeg,*

*Manitoba, R3T 2N2 Canada*

Contents

[Ultrashort Cationic Lipopeptides and Lipopeptoids Selectively Induce Cytokine Production in monocytic cells. 1](#__RefHeading___Toc342554548)

[General Chemical Procedures 2](#__RefHeading___Toc342554549)

[t-Butyl (4-aminobutyl)carbamate Synthesis 2](#__RefHeading___Toc342554550)

[Fmoc Deprotection 3](#__RefHeading___Toc342554551)

[Amino Acid Coupling 3](#__RefHeading___Toc342554552)

[Peptoid residue synthesis 3](#__RefHeading___Toc342554553)

[Carboxylic Acid Coupling 4](#__RefHeading___Toc342554554)

[Cleavage from Rink Amide MBHA Resin 4](#__RefHeading___Toc342554555)

[Purification of Lipopeptides and Lipopeptoids 5](#__RefHeading___Toc342554556)

[Guanidinylation Reaction 5](#__RefHeading___Toc342554557)

[Spectral data for newly synthesized compounds. 6](#__RefHeading___Toc342554558)

[Weakly Active Amphiphiles 12](#__RefHeading___Toc342554559)

[Immunomodulatory Properties 12](#__RefHeading___Toc342554560)

[Antimicrobial Activity of Select Amphiphiles 12](#__RefHeading___Toc342554561)

[Supplementary Materials References 12](#__RefHeading___Toc342554562)

[Figures and Tables 13](#__RefHeading___Toc342554563)

## General Chemical Procedures

Reagents and solvents were purchased from commercially available sources and used without purification, unless otherwise noted. Fluorinated carboxylic acids were purchased from Fluorous Technologies Incorporated. Flash chromatography was performed using silica gel (Silicycle 23 -60 um) using standard techniques. ^1^H and ^13^C NMR were recorded on a Bruker AMX-500 or Bruker AMX-300 spectrometer in the noted solvents. Chemical shifts (δ) are reported in parts per million relative to tetramethylsilane. Compounds were visualized with either a mix of ninhydrin and acetic acid in ethanol, after spotting onto glass backed TLC plates. Low-resolution mass spectra (ESI+) were obtained on a Varian 500-MS IT Mass Spectrometer. All compounds were determined to be ≥90% pure by NMR prior to bacterial testing. Rink Amide MBHA resin was used to prepare each peptide and was swelled in DMF for a minimum of thirty minutes prior to initial deprotection.

### t-Butyl (4-aminobutyl)carbamate Synthesis

To a rapidly stirring solution of 1,4-diaminobutane (34.0 mL, 3 eq) and triethylamine (25.0 mL) in ice cold methanol (225 mL) was added a solution of Boc anhydride (24.9 g, 1 eq) in methanol (50 mL) over a period of 1 hr. The solution was allowed to gradually warm to room temperature and stir overnight. The methanol and triethylamine was then removed, and water added. Acetic acid was added (6.5 mL, 1.1 eq), and the solution extracted twice with diethyl ether. The aqueous layer was then basified with sodium carbonate and extracted twice with DCM. The DCM layers were combined, washed with 10% NaCO_3_, and concentrated. t-Butyl (4-aminobutyl)carbamate was obtained as a light yellow oil and used without further purification (10.4g, 49%).

### Fmoc Deprotection

To remove the Fmoc protecting group, DMF:Piperidine (4:1) was added to pre-swelled resin, until it reached a level approximately three times the height of the bead bed, and the beads were gently agitated by a steady stream of air for forty minutes. The DMF:Piperidine mixture was then drained and the deprotecting process was repeated. The beads were then washed successively three times with DMF, then DCM, then DMF again, and a small sample of the beads was removed. This sample was treated with equal volumes of 2% chloranil and 2% acetaldehyde in DMF and successful deprotection was observed by the beads turning bright red.

### Amino Acid Coupling

To freshly deprotected beads was added a solution containing the Fmoc protected amino acid derivative (3 equivalents), TBTU (3 equivalents) and Hunig’s Base (8 equivalents) in DMF. The mixtures were premixed at least three minutes prior to addition to ensure effective activation of the carboxylic acids. The solution was then gently agitated for at least three hours, after which time the solvent was drained and the beads were successively washed with 3x DMF, DCM and DMF. Completion of the reaction was verified through the chloranil test, as successfully reacted beads would not change colour in the presence of equal quantities of 2% chloranil and 2% acetaldehyde in DMF.

### Peptoid residue synthesis

Following established procedure, ^1^ to the Rink Amide peptoid containing resin was added diisopropylcarbodiimide (DIC) (16.6 equivalents) and 2-bromoacetic acid (20 equivalents) in DMF. The mixture was agitated with a constant flow of N_2_ gas for thirty minutes, during which time a light yellow foam developed. The reaction mixture was then removed via filtration, and the resin washed three times successively with DMF, DCM and DMF. A solution of tert-butyl (4-aminobutyl)carbamate (20 equivalents) in NMP was then added and the beads once more agitated by nitrogen gas, now for ninety minutes. Once again the reaction mixture was filtered away and the beads were washed three times successively with NMP, DCM and DMF. Complete reaction of free amines was verified through a chloranil test, and the synthesis was carried forward with new residues as appropriate.

### Carboxylic Acid Coupling

As in the elongation of the amino acid chain, hydrophobic tails were attached to the resin through the use of a mixture containing the carboxylic acid (3 equivalents), TBTU (3 equivalents) and Hunig’s Base (8 equivalents) in DMF. Over the course of the syntheses it was found that activated, fluorous carboxylic acids have extremely poor solubility in DMF, leading to the formation of a thick gel. As a result, these compounds were instead premixed in DCM, and Pybop (3 equivalents) was used as the activating agent. The quantity of Hunig’s base was unchanged (8 equivalents). Once the coupling mix was added the beads were gently agitated for approximately three hours, after which the chloranil test demonstrated that the coupling was complete.

### Cleavage from Rink Amide MBHA Resin

Resin containing the fully protected, complete amino acids was rinsed three times with DCM to remove any residual DMF and dried. Residual DMF was found to interact with the TFA, forming a liquid which could not be removed even under low pressure (>0.001 Torr) using a dry-ice equipped rotary evaporator. An acidic solution of TFA:Water:TIPS (95:2.5:2.5) was then added and the beads were agitated for a minimum of two hours. The TFA was then filtered off and concentrated via evaporation under reduced pressure to yield the crude lipopeptides.

### Purification of Lipopeptides and Lipopeptoids

Peptides were taken up in a minimum quantity of water and loaded onto a column containing reverse phase flash silica. The peptides were then eluted by washing the column successively with distilled water (2.5 CV), 50% MeOH in water (2 CV), 75% MeOH in water (2 CV) and MeOH (3 CV). All solvents were stored in glass bottles, and acidified to a concentration of 0.1% TFA. After three or more peptides had been purified with the column the column was washed with DCM and 1% TFA in MeOH. Fractions containing the peptides of interest were collected and the solvent was removed via the steady passage of air at atmospheric pressure.

### Guanidinylation Reaction

Following established procedure,^2^ full length peptides and peptoids of interest were dissolved in a mixture of 1,4-dioxane and water. Nˈ,Nˈˈ-diboc-N-triflylguanidine (3 eq per amine) and triethylamine (1.5 eq per amine) were then added, and the mixture was stirred at room temperature for 3-4 days. The dioxane was then removed under reduced pressure and the mixture was extracted three times with CHCl_3_. The organic layer was washed once with brine and dried over anhydrous sodium sulfate to yield the crude NBoc protected peptides and peptoids. Flash chromatography in 9:1 DCM:MeOH provided the pure Nboc protected amphiphiles as white solids.

Cleavage of the Boc protecting groups was effected by stirring the compounds in 9:1 TFA:H_2_O for one hour. Residual polar functionalities were removed via trituration in 49:1 Et_2_O:MeOH to give the desired compounds as clear oils.

## Spectral data for newly synthesized compounds.

**C16-LysGlyLys (1)**

^1^H NMR (500 MHz, MeOD) δ 4.34 (dd, J = 9.6, 4.7, 1H), 4.22 (dd, J = 8.1, 6.2, 1H), 3.96 – 3.81 (m, 2H), 3.01 – 2.84 (m, 4H), 2.33 – 2.17 (m, 2H), 1.97 – 1.38 (m, 14H), 1.38 – 1.21 (m, 24H), 0.90 (t, J = 7.0, 3H). ^13^C NMR (126 MHz, MeOD) δ 176.9, 176.8, 175.4, 171.7, 55.3, 54.3, 43.8, 40.5, 40.5, 36.7, 33.1, 32.2, 31.9, 30.8, 30.8, 30.7, 30.5, 30.5, 30.4, 28.1, 27.9, 26.8, 23.8, 23.8, 23.7, 14.5. MS (ES) Calc. for C_30_H_61_N_6_O_4_ (M+H)^+^: 569.5. Found 569.7.

**C16OH-LysGlyLys (2)**

^1^H NMR (500 MHz, MeOD, mixture of rotamers) δ 4.38 – 4.27 (m, 2H), 4.19 (dd, J = 8.1, 6.2, 1H), 3.93 – 3.79 (m, 2H), 3.50 (t, J = 6.7, 1H), 2.95 – 2.83 (m, 4H), 2.29 – 2.17 (m, 2H), 1.93 – 1.18 (m, 38H). ^13^C NMR (126 MHz, MeOD) δ 177.0, 176.9, 175.5, 171.8, 63.2, 55.4, 54.4, 43.9, 40.6, 40.6, 36.8, 33.8, 32.4, 32.0, 31 – 30.5 m, aliphatic carbons), 30.3, 29.3, 28.2, 28.0, 27.1, 26.9, 26.8, 23.9, 23.9. MS (ES) Calc. for C_30_H_61_N_6_O_5_ (M+H)^+^: 585.5. Found 585.7.

**C20-LysGlyLys (3)**

^1^H NMR (500 MHz, MeOD) δ 4.42 – 4.30 (m, 1H), 4.22 (dd, J = 8.2, 6.1, 1H), 3.89 (q, J = 16.7, 2H), 3.00 – 2.86 (m, 4H), 2.26 (td, J = 7.4, 3.2, 2H), 1.97 – 1.38 (m, 14H), 1.38 – 1.21 (m, 32H), 0.95 – 0.84 (m, 3H), 0.00 (dd, J = 8.2, 6.1, 1H), 4.39 – 4.29 (m, 1H), 3.97 – 3.81 (m, 2H), 2.34 – 2.19 (m, 2H), 3.02 – 2.83 (m, 4H).  ^13^C NMR (126 MHz, MeOD) δ 176.8, 176.8, 175.3, 171.7, 55.2, 54.2, 43.7, 40.5, 40.5, 36.7, 33.1, 32.3, 31.9 – 30.4 (m, aliphatic carbons), 28.1, 27.9, 26.8, 23.8, 23.8, 23.7, 14.5. MS (ES) Calc. for C_34_H_69_N_6_O_4_ (M+H)^+^: 625.5. Found 625.8.

**C11-HarHarHar NBoc**

^1^H NMR (500 MHz, CDCl3) δ 11.46 (s, 3H), 8.33 (d, J = 4.1, 3H), 4.47 – 4.19 (m, 3H), 3.54 – 3.24 (m, 6H), 2.23 (t, J = 7.7, 2H), 2.01 – 1.15 (m, 88H), 0.86 (t, J = 7.0, 3H). ^13^C NMR (75 MHz, CDCl3) δ 174.6, 173.9, 173.2, 172.7, 171.6, 156.2, 156.1, 156.1, 153.3, 153.2, 83.6, 83.4, 83.3, 54.2, 53.0, 41.1, 40.8, 40.8, 40.7, 36.5, 32.0, 29.7 – 29.4 (m, aliphatic peaks), 28.6, 28.6, 28.3, 28.1, 28.0, 25.6, 23.1, 22.7, 14.1.

**C11-HarHarHar (8)**

^1^H NMR (500 MHz, D2O) δ 4.33 – 4.14 (m, 3H), 3.17 – 3.04 (m, 6H), 2.23 (t, J = 6.9, 2H), 1.86 – 1.27 (m, 20H), 1.27 – 1.13 (m, 14H), 0.79 (t, J = 6.8, 3H). ^13^C NMR (126 MHz, D2O) δ 177.5, 176.4, 174.3, 173.7, 156.7, 156.7, 156.7, 53.6, 53.4, 53.4, 40.9, 40.9, 40.8, 35.3, 31.2, 30.5, 30.4, 30.3, 28.7, 28.5, 28.4, 28.1, 27.3, 25.3, 22.3, 22.2, 22.1, 22.1, 13.4. MS (ES) Calc. for C_32_H_66_N_13_O_4_ (M+H)^+^: 696.5. Found 696.7.

**C16-HarHarHar NBoc**

^1^H NMR (500 MHz, CDCl3) δ 11.45 (s, 3H), 8.37 (s, 3H), 4.48 – 4.21 (m, 3H), 3.50 – 3.29 (m, 6H), 2.25 (t, J = 7.6, 2H), 2.02 – 1.15 (m, 98H), 0.87 (t, J = 6.9, 3H). ^13^C NMR (75 MHz, CDCl3) δ 174.3, 173.8, 171.5, 156.1, 156.1, 156.1, 153.2, 83.4, 83.3, 83.2, 55.7, 54.0, 52.9, 40.7, 36.4, 31.9, 31.5, 31.4, 31.4, 29.7 – 27.9 (m, aliphatic peaks), 25.6, 23.1, 22.7, 14.1.

**C16-HarHarHar (9)**

^1^H NMR (500 MHz, D2O) δ 4.42 – 4.20 (m, 3H), 3.32 – 3.08 (m, 6H), 2.36 – 2.21 (m, 2H), 1.98 – 1.36 (m, 20H), 1.29 (s, 24H), 0.89 (t, J = 6.6, 3H). ^13^C NMR (126 MHz, D2O) δ 176.3, 174.2, 173.6, 156.7, 156.7, 156.7, 53.8, 53.5, 53.4, 40.9, 40.8, 35.4, 31.5, 30.6, 30.5, 29.4 - 28.7 (m, aliphatic peaks), 27.5, 27.4, 27.4, 25.4, 22.4, 22.3, 22.3, 22.2, 13.6. MS (ES) Calc. for C_37_H_76_N_13_O_4_ (M+H)^+^: 766.6. Found 766.7.

**C16OH-HarHarHar NBoc**

^1^H NMR (300 MHz, CDCl3) δ 11.44 (s, 3H), 8.30 (s, 3H), 4.59 – 4.22 (m, 3H), 3.61 (t, J = 6.6, 2H), 3.36 (s, 6H), 2.35 – 2.08 (m, 2H), 2.02 – 1.09 (m, 100H). ^13^C NMR (75 MHz, CDCl3) δ 174.3, 173.9, 172.5, 171.5, 163.5, 163.4, 163.3, 156.2, 156.2, 156.1, 153.3, 83.3, 83.3, 83.2, 79.6, 79.5, 79.4, 63.1, 53.9, 53.8, 52.9, 40.8, 40.7, 40.7, 36.5, 32.9, 31.9, 31.6, 29.6, 29.5, 29.5, 29.4, 28.8, 28.7, 28.6, 28.4, 28.3, 28.2, 28.1, 25.8, 25.7, 23.1, 23.1.

**C16OH-HarHarHar (10)**

^1^H NMR (500 MHz, D2O) δ 4.43 – 4.24 (m, 3H), 3.61 (t, J = 6.7, 2H), 3.26 – 3.09 (m, 6H), 2.39 – 2.17 (m, 2H), 1.95 – 1.17 (m, 44H). ^13^C NMR (126 MHz, D2O) δ 177.0, 174.8, 174.3, 157.4, 157.4, 157.4, 62.5, 54.4, 54.2, 54.0, 41.5, 41.5, 36.1, 32.3, 31.2, 31.2, 31.1, 31.1, 30.2, 30.2, 29.9 – 29.6 (m, aliphatic peaks), 29.4, 29.2, 28.1, 28.1, 28.0, 28.0, 26.1, 26.0, 26.0, 23.0, 23.0, 22.9, 22.9, 22.8, 22.8. MS (ES) Calc. for C_37_H_76_N_13_O_5_ (M+H)^+^: 782.6. Found 782.9.

**C20-HarHarHar (11)**

^1^H NMR (500 MHz, D2O) δ 4.43 – 4.19 (m, 3H), 3.30 – 3.07 (m, 6H), 2.44 – 2.15 (m, 2H), 1.98 – 1.51 (m, 14H), 1.51 – 1.09 (m, 32H), 0.90 (t, J = 6.2, 3H). ^13^C NMR (126 MHz, D2O) δ 176.2, 175.9, 174.1, 173.5, 156.7, 156.7, 156.7, 53.8, 53.6, 53.5, 40.8, 35.6, 31.9, 30.7, 29.8 – 29.6 (m, aliphatic peaks), 29.4, 29.2, 27.7, 27.5, 25.6, 22.6, 22.3, 13.8. MS (ES) Calc. for C_41_H_85_N_13_O_4_ (M+2H)^2+^: 411.8. Found 412.1.

**C16OH-NlysNlysNlys (16)**

^1^H NMR (500 MHz, D2O, mixture of rotamers) δ 4.54 – 3.92 (m, 6H), 3.55 (t, J = 6.9, 2H), 3.50 – 3.21 (m, 6H), 3.08 – 2.90 (m, 6H), 2.48 – 2.33 (m, 1H), 2.27 – 2.10 (m, 1H), 1.89 – 1.46 (m, 16H), 1.28 (s, 22H). ^13^C NMR (126 MHz, D2O) δ 177.1, 177.1, 176.8, 176.7, 176.1, 173.5, 173.4, 173.0, 172.8, 172.5, 172.4, 172.1, 170.9, 170.9, 170.6, 170.4, 170.3, 170.2, 170.1, 170.0, 170.0, 169.9, 169.8, 169.7, 169.3, 61.9, 49.8, 49.5, 49.4, 49.4, 49.2, 49.2, 49.0, 48.8, 48.8, 48.7, 48.3, 48.2, 48.2, 48.1, 48.0, 47.9, 47.7, 47.7, 47.6, 47.5, 47.3, 47.3, 47.1, 47.0, 46.9, 46.8, 46.8, 39.1, 39.0, 33.0, 32.9, 32.5, 32.4, 31.9, 31.8, 29.5, 29.4, 29.4, 29.3, 29.2, 29.0, 28.9, 28.8, 25.6, 25.5, 25.2, 25.1, 25.0, 25.0, 24.9, 24.9, 24.8, 24.8, 24.7, 24.6, 24.5, 24.1, 24.1, 23.8, 23.8, 23.7, 23.6, 23.5. MS (ES) Calc. for C_38_H_78_N_7_O_4_ (M+H)^+^: 656.5. Found 656.7.

**C16OH-N_Har_N_Har_N_Har_ NBoc**

^1^H NMR (300 MHz, CDCl3) δ 11.50 (s, 3H), 8.38 (s, 3H), 4.08 (d, J = 42.6, 6H), 3.62 (dd, J = 7.0, 6.2, 2H), 3.44 (s, 12H), 2.32 (t, J = 7.3, 2H), 1.83 – 1.04 (m, 92H). ^13^C NMR (75 MHz, CDCl3) δ 173.6, 171.2, 169.7, 169.1, 156.3, 156.3, 156.3, 153.4, 153.3, 83.4, 83.4, 83.3, 79.6, 79.6, 79.5, 63.1, 40.5, 32.9, 29.7, 29.6, 29.6, 29.5, 28.4, 28.2, 26.6, 26.6, 26.6, 26.5, 26.1, 25.9, 25.3.

**C16OH-*N*Har*N*Har*N*Har (20)**

^1^H NMR (500 MHz, D2O, mixture of rotamers) δ 4.57 – 4.02 (m, 6H), 3.64 – 3.51 (m, 2H), 3.51 – 3.28 (m, 6H), 3.28 – 2.98 (m, 6H), 2.55 – 2.35 (m, 1H), 2.27 – 2.12 (m, 1H), 1.89 – 1.49 (m, 16H), 1.49 – 1.14 (m, 22H). ^13^C NMR (126 MHz, D2O, mixture of rotamers) δ 176.0, 173.5, 173.4, 171.1, 170.9, 170.3, 170.1, 156.8, 156.8, 156.8, 61.9, 49.2, 48.4, 47.5, 40.8, 40.8, 32.8, 32.4, 31.8, 31.7, 29.8 – 28.6 (m, aliphatic peaks), 29.4, 29.2, 29.2, 29.0, 25.5 – 25.1 (m, aliphatic peaks), 23.9.

**C7-LysGlyLys (S1)**

^1^H NMR (300 MHz, MeOD) δ 4.44 – 4.15 (m, 2H), 4.02 – 3.80 (m, 2H), 3.07 – 2.83 (m, 4H), 2.40 – 2.16 (m, 2H), 2.04 – 1.22 (m, 20H), 0.92 (t, J = 6.6, 3H). ^13^C NMR (75 MHz, MeOD) δ 177.0, 176.9, 175.5, 171.8, 55.4, 54.4, 43.9, 40.6, 40.6, 36.8, 32.8, 32.4, 32.0, 30.2, 28.2, 28.0, 26.9, 23.9, 23.9, 23.7, 14.5. MS (ES) Calc. for C_21_H_43_N_6_O_4_ (M+H)^+^: 443.3. Found 443.4.

**C9B-LysGlyLys (S2)**

^1^H NMR (300 MHz, MeOD) δ 4.37 (dd, J = 9.4, 4.8, 1H), 4.24 (dd, J = 7.9, 6.3, 1H), 3.98 – 3.82 (m, 2H), 3.07 – 2.84 (m, 4H), 2.28 (t, J = 7.3, 2H), 2.04 – 1.06 (m, 23H), 0.90 (d, J = 6.6, 6H). ^13^C NMR (75 MHz, MeOD) δ 176.8, 176.6, 174.9, 174.2, 55.1, 54.8, 54.3, 40.6, 40.6, 36.9, 32.8, 32.3, 32.3, 30.9, 30.6, 29.3, 28.6, 28.2, 28.2, 28.1, 27.1, 24.0, 23.9, 23.8, 23.2. MS (ES) Calc. for C_21_H_49_N_6_O_4_ (M+H)^+^: 485.4. Found 485.4.

**F9B-LysGlyLys (S3)**

^1^H NMR (300 MHz, MeOD) δ 4.37 (dd, J = 9.4, 4.7, 1H), 4.27 (dd, J = 7.9, 6.2, 1H), 4.01 – 3.83 (m, 2H), 3.03 – 2.84 (m, 4H), 2.75 – 2.38 (m, 4H), 2.00 – 1.33 (m, 12H). ^13^C NMR (75 MHz, MeOD) δ 176.9, 175.2, 173.4, 171.8, 55.5, 54.2, 43.9, 40.6, 40.6, 32.4, 32.1, 28.3, 28.0, 27.8, 27.5 - 27.3 (m, adjacent to CF bonds), 23.9. MS (ES) Calc. for C_24_H_34_F_15_N_6_O_4_ (M+H)^+^: 755.2. Found 755.2.

**F9-LysLysLys (S4)**

^1^H NMR (300 MHz, MeOD) δ 4.48 – 4.20 (m, 3H), 2.94 (dd, J = 8.4, 4.6, 6H), 2.68 – 2.37 (m, 4H), 2.02 – 1.29 (m, 18H). ^13^C NMR (75 MHz, MeOD) δ 176.7, 174.8, 174.3, 173.3, 55.3, 54.9, 54.2, 40.6, 40.6, 40.6, 32.7, 32.3, 32.2, 28.2, 28.1, 28.1, 27.8, 27.5 – 27.4 (m, adjacent to CF bonds), 23.9, 23.9, 23.8. MS (ES) Calc. for C_27_H_43_F_13_N_7_O_4_ (M+H)^+^: 776.3. Found 775.6.

**F7-HarGHar (S5)**

^1^H NMR (500 MHz, MeOD) δ 4.43 – 4.30 (m, 1H), 4.30 – 4.15 (m, 1H), 3.98 – 3.78 (m, 2H), 3.27 – 3.07 (m, 4H), 2.69 – 2.42 (m, 4H), 1.98 – 1.80 (m, 2H), 1.80 – 1.67 (m, 2H), 1.67 – 1.26 (m, 8H). ^13^C NMR (126 MHz, MeOD) δ 176.9, 175.2, 173.2, 171.7, 158.7, 158.5, 66.9, 55.5, 54.2, 49.9, 43.7, 42.2, 32.4, 32.0, 29.4, 29.2, 27.5 (t, J=21.8), 27.1 (t, J=5.7), 23.9, 23.9, 15.4. MS (ES) Calc. for C_23_H_38_F_9_N_10_O_4_ (M+H)^+^: 689.29. Found 689.6.

## Weakly Active Amphiphiles

See Table S1

### Immunomodulatory Properties

See Figure S1 and Figure S2.

## Antimicrobial Activity of Select Amphiphiles^3, 4^

See Table S2

## Supplementary Materials References

1. Chongsiriwatana, N. P.; Patch, J. A.; Czyzewski, A. M.; Dohm, M. T.; Ivankin, A.; Gidalevitz, D.; Zuckermann, R. N.; Barron, A. E. Peptoids that mimic the structure, function, and mechanism of helical antimicrobial peptides. *Proc. Natl. Acad. Sci. U. S. A.* **2008**, *105,* 2794-2799.

2. Baker, T. J.; Luedtke, N. W.; Tor, Y.; Goodman, M. Synthesis and anti-HIV activity of guanidinoglycosides. *J. Org. Chem.* **2000**, *65,* 9054-9058.

3. Findlay, B.; Szelemej, P.; Zhanel, G. G.; Schweizer, F. Guanidylation and Tail Effects in Cationic Antimicrobial Lipopeptoids. *PloS one* **2012**, *7,* e41141.

4. Findlay, B.; Zhanel, G. G.; Schweizer, F. Investigating the Antimicrobial Peptide “Window of Activity” using Cationic Lipopeptides with Hydrocarbon and Fluorinated Tails. *Int. J. Antimicrob. Agents* **2012**, *40,* 36--42.

## Figures and Tables

**Figure S1.** Immunological properties of the compounds presented in table S1. Human macrophage-like THP-1 cells were exposed to amphiphiles **S1-S7** for twenty-four hours. Tissue culture (TC) supernatants were monitored for A) IL-8 production and B) Gro-alpha production by ELISA. IL-8 production is shown after subtraction of constitutive background levels found in un-stimulated control cell. C) LDH release was monitored in the TC supernatants as an indicator of cellular cytotoxicity. Results shown represent percent cytotoxicity over un-stimulated cells. Studies were performed in duplicate, with the data here presented as the mean plus standard error of the mean (sem).

**Figure S2.** TNF-α production by human macrophage-like THP-1 cells following incubation with amphiphiles **1-21**. TC supernatants were monitored for cytokine production via ELISA, and results are reported in pg/mL. All studies were performed in two independent biological replicates with two technical replicates each.

**Table S1.** Compound sequences.

^a^ Trifluroacetate salt

**Table S2.** Antimicrobial activity of select amphiphiles, derived from previous research.

^a^ Values are given in μM.
